# Flux measurements and maintenance energy for carbon dioxide utilization by *Methanococcus maripaludis*

**DOI:** 10.1186/s12934-015-0336-z

**Published:** 2015-09-16

**Authors:** Nishu Goyal, Mrutyunjay Padhiary, Iftekhar A. Karimi, Zhi Zhou

**Affiliations:** Department of Chemical and Biomolecular Engineering, National University of Singapore, 4 Engineering Drive 4, Singapore, 117585 Singapore; Department of Civil and Environmental Engineering, National University of Singapore, 1 Engineering Drive 2, Singapore, 117576 Singapore; Division of Environmental and Ecological Engineering and School of Civil Engineering, Purdue University, 550 Stadium Mall Drive, West Lafayette, IN 47907 USA

**Keywords:** *M. maripaludis*, Hydrogenotrophic methanogen, CO_2_ utilization, Maintenance energy, Extracellular fluxes

## Abstract

**Background:**

The rapidly growing mesophilic methanogen *Methanococcus maripaludis* S2 has a unique ability to consume both CO_2_ and N_2_, the main components of a flue gas, and produce methane with H_2_ as the electron donor. The existing literature lacks experimental measurements of CO_2_ and H_2_ uptake rates and CH_4_ production rates on *M. maripaludis*. Furthermore, it lacks estimates of maintenance energies for use with genome-scale models. In this paper, we performed batch culture experiments on *M. maripaludis* S2 using CO_2_ as the sole carbon substrate to quantify three key extracellular fluxes (CO_2_, H_2_, and CH_4_) along with specific growth rates. For precise computation of these fluxes from experimental measurements, we developed a systematic process simulation approach. Then, using an existing genome-scale model, we proposed an optimization procedure to estimate maintenance energy parameters: growth associated maintenance (GAM) and non-growth associated maintenance (NGAM).

**Results:**

The measured extracellular fluxes for *M. maripaludis* showed excellent agreement with in silico predictions from a validated genome-scale model (*i*MM518) for NGAM = 7.836 mmol/gDCW/h and GAM = 27.14 mmol/gDCW. *M. maripaludis* achieved a CO_2_ to CH_4_ conversion yield of 70–95 % and a growth yield of 3.549 ± 0.149 g DCW/mol CH_4_ during the exponential phase. The ATP gain of 0.35 molATP/molCH_4_ for *M. maripaludis*, computed using NGAM, is in the acceptable range of 0.3–0.7 mol ATP/molCH_4_ reported for methanogens. Interestingly, the uptake distribution of amino acids, quantified using *i*MM518, confirmed alanine to be the most preferred amino acids for growth and methanogenesis.

**Conclusions:**

This is the first study to report experimental gas consumption and production rates for the growth of *M. maripaludis* on CO_2_ and H_2_ in minimal media. A systematic process simulation and optimization procedure was successfully developed to precisely quantify extracellular fluxes along with cell growth and maintenance energy parameters. Our growth yields, ATP gain, and energy parameters fall within acceptable ranges known in the literature for hydrogenotrophic methanogens.

## Background

In light of rapidly growing CO_2_ emissions, capture and conversion of CO_2_ to useful fuels and chemicals is becoming increasingly important. Since the redox potential of converting CO_2_ to CH_4_ is high (−240 mV), a synthetic chemical conversion requires expensive catalysts, harsh reaction conditions, and high energy [1]. In contrast, coenzymes in methanogens can act as biocatalysts and reduce the activation energy for higher conversion efficiency under benign conditions. Thus, a microbial conversion process not only captures CO_2_ from flue gas emissions, but also makes a promising carbon–neutral biofuel, such as CH_4_.

The ecological role of methanogens to remove CO_2_ from the environment via methanogenesis has been widely studied [2, 3]. Among methanogens, *M. maripaludis* S2 is a fully sequenced, rapidly growing, hydrogenotrophic methanogen, that has the capability to consume major components (CO_2_ and N_2_) of a flue gas [4, 5]. *M. maripaludis* converts CO_2_ to CH_4_ in the presence of electron donors such as H_2_ [4] or formate [6], and also possesses a unique ability to fix N_2_ to ammonia [5, 7, 8]. Although several studies have characterized and engineered the metabolic pathways in *M. maripaludis*, quantitative measurements on CO_2_ utilization and CH_4_ production are absent in the literature. Furthermore, no study to date has quantified the distribution of carbon flux between biomass synthesis and methanogenesis in this microorganism.

Genome-scale models are very useful for quantifying extracellular and intracellular fluxes, analyzing cultivation data, designing media and processes, and engineering microbial strains for enhanced production [9, 10]. However, these models need to be validated with experimental flux measurements to accurately predict intracellular metabolic fluxes [11]. While extracellular fluxes can be measured by estimating changes in external metabolite concentrations, intracellular flux measurements are difficult because ^13^C NMR labeling is usually required [12]. We developed a genome-scale metabolic model (*i*MM518) for *M. maripaludis* S2 [13], but the model had not been fully validated due to inadequate quantitative data on uptake and production rates.

In this study, we performed batch culture experiments and quantified three key extracellular fluxes (CO_2_, H_2_, and CH_4_) and specific growth rates of *M. maripaludis*. To the best of our knowledge, this is the first experimental study to report CO_2_, H_2_ consumption and CH_4_ production rates with CO_2_ as the sole carbon source. In addition, this study presents novel approaches to quantify extracellular fluxes and determine maintenance energy parameters using experimentally measured extracellular fluxes along with a genome-scale model. Using the model, we analyzed the effects of amino acids on growth rates, CO_2_ utilization rates, and CH_4_ production rates, and studied the distribution of carbon flux between biomass synthesis and methanogenesis.

## Results and discussion

### Cell growth

**Fig. 1:**
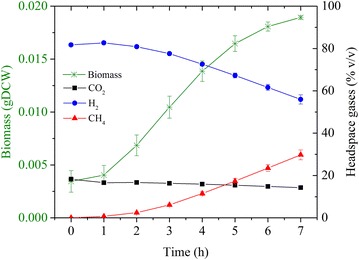
Profiles of headspace gas compositions and biomass of *M. maripaludis* S2 in batch cultures under minimal media conditions using CO_2_ as the sole carbon substrate. *gDCW* gram dry cell weight

*Methanococcus maripaludis* grew extremely well on CO_2_ without any complex substrates, such as acetate and yeast extract. The measured cell growth profile for *M. maripaludis* is shown in Fig. 1. The dry cell biomass increased by 15.49 mg in 7 h. The doubling time was about 2 h, which is consistent with the literature [4]. The lag phase duration varied with the state of inoculum, and found to be the shortest for an inoculum from the late exponential phase (data not shown). Figure 1 also shows the concentration profiles (% v/v) of CO_2_, H_2_, and CH_4_ in the headspace of the reactor over a period of about 7 h. The headspace pressure dropped from 250 to 100 kPa. The headspace contained 80/20 v/v H_2_/CO_2_ at time zero. The metabolic/biocatalytic action of *M. maripaludis* increased methane concentration in the headspace to approximately 30 % v/v at the end of 7 h.

**Fig. 2:**
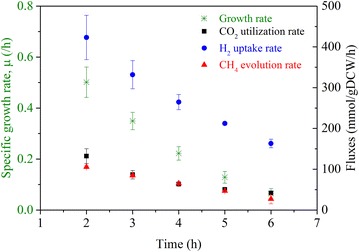
The time profiles of specific growth rates and corresponding extracellular fluxes (CO_2_, H_2_, and CH_4_)

As the headspace pressure decreased with time, both growth rates and extracellular fluxes decreased (Fig. 2). The maximum growth rate was estimated to be 0.50 ± 0.05/h for a CO_2_ uptake of 132.13 ± 15.13 mmol/gDCW/h, H_2_ uptake of 423.06 ± 44.94 mmol/gDCW/h, and CH_4_ production of 105.61 ± 17.75 mmol/gDCW/h. Kral et al. [14] reported a H_2_ uptake of 28.8 mmol/gDCW/h in inorganic media. However, they did not state the growth phase for this rate. Our observed H_2_ uptake was 423.06 ± 44.94 mmol/gDCW/h in the early exponential phase, and 107.5 ± 44.94 mmol/gDCW/h at the end of 7 h, suggesting that the rate reported by Kral et al. [14] might be measured for a late exponential phase. Lupa et al. [6] reported methane evolution rates (MERs) ranging from 9.40 to 27.55 mmol/gDCW/h for cell growth rates of 0.04–0.13/h, which is close to our MER of 27.19 ± 17.75 mmol/gDCW/h for a growth rate of 0.064 ± 0.049/h in the late exponential phase. Apart from these two studies, no other data have been reported in the literature for the uptake and production rates of *M. maripaludis*. Thus, our study is the first to give a full range of comprehensive growth and flux data for *M. maripaludis*. In fact, we could not find similar data for any other methanogen except for one study [15] on *M. barkeri*, which reported a maximum H_2_ uptake rate of 41 mmol/gDCW/h with a corresponding CO_2_ uptake rate of 11.61 mmol/gDCW/h and CH_4_ production rate of 8.82 mmol/gDCW/h. Our observed fluxes are one order of magnitude higher than those reported for *M. barkeri*, which could be attributed to the doubling time of *M. maripaludis* (~2 h) being much shorter than that of *M. barkeri* (~30 h).

From the plot of dry cell weight (g) versus methane produced over time, we obtained a growth yield of 3.549 ± 0.149 gDCW/molCH_4_ for *M. maripaludis* during the exponential phase. Table 1 shows a comparison of growth yield and specific growth rate for different methanogens. The yield of *M. maripaludis* matches well with the yield reported for other hydrogenotrophic methanogens growing on H_2_/CO_2_ in batch cultures [16, 17]. Although a much higher yield on H_2_/CO_2_ (8.7 ± 0.8 gDCW/mol CH_4_) was reported in *M. barkeri* [18], the specific growth rate of *M. maripaludis* observed in this study (0.346/h) was 5.97-fold higher than that in *M. barkeri* (0.058/h) [18]. A high specific growth rate suggests that *M. maripaludis* can grow rapidly and have good potentials for industrial and environmental applications.

**Table 1:** Quantitative comparison of growth yields and specific growth rates for some methanogens

Organism	Substrate	Yield, Y_CH4_ (gDCW/molCH_4_)	Specific growth rate, µ(/h)	References
*M. thermoautotrophicum*	H_2_, CO_2_	1.6–3	0.690	[16]
*M. bryantii*	H_2_, CO_2_, organic supplements	2.4	0.031	[32]
*M. str AZ*	H_2_, CO_2_	2.32	0.110	[33]
*M. barkeri*	H_2_, CO_2_	8.7	0.058	[18]
*M. formicicum*	H_2_, CO_2_, organic supplements	3.5	0.060	[34]
*M. maripaludis*	H_2_, CO_2_	3.54	0.346	This study

*gDCW* gram dry cell weight

### Extracellular fluxes

**Fig. 3:**
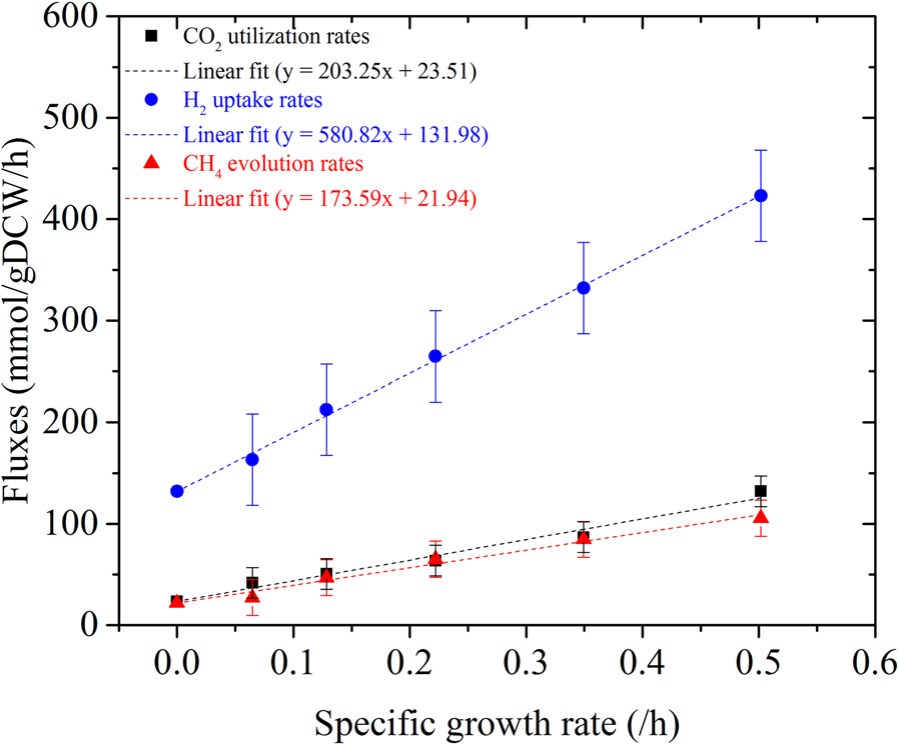
Linear correlations between extracellular fluxes and specific growth rates

Using the data from Fig. 2, the extracellular fluxes (CO_2_, H_2_, and CH_4_) are correlated linearly with specific growth rates in Fig. 3. Using these linear correlations, we obtained the following relations among the extracellular fluxes:

1 $$v_{{{\text{H}}_{ 2} }} /v_{{{\text{CO}}_{ 2} }} = 2.858 + 64.759/v_{{{\text{CO}}_{ 2} }}$$

2 $$v_{{{\text{CH}}_{ 4} }} /v_{{{\text{CO}}_{ 2} }} = 0.854 + 1.855/v_{{{\text{CO}}_{ 2} }}$$

Equation 2 suggests that the fraction of CO_2_ conversion to CH_4_ decreases with increase in CO_2_ uptake rate (or equivalently cell growth). This is consistent with the fact that cell growth competes with methanogenesis for carbon utilization [13]. When cell growth is low, most of the carbon is diverted to produce energy for maintenance via methanogenesis, resulting in a higher CH_4_ yield, and vice versa. Our observed conversion fraction for maximum growth rate is 0.868, which is higher than 0.810 reported for *M. barkeri* in a chemostat culture grown on H_2_/CO_2_ [19].

Gas-to-liquid mass transfer of O_2_, CO_2_, H_2_, N_2_ etc. plays an important role in the cultivation of microbes [20]. Various factors such as gas–liquid interfacial area, mixing, temperature, and pressure affect this mass transfer. Thus, we expected higher uptake rates of CO_2_ and H_2_ in *M. maripaludis* with enhanced mass transfer. To confirm this, we tested the effect of mixing and gas–liquid interfacial area on the growth and metabolism of *M. maripaludisS2* at 37 °C. Increasing the gas–liquid interfacial area by positioning the bottle reactor from vertical to horizontal roughly doubled MER, while shaking the reactor increased it multiple folds (data not shown).

### GAM, NGAM, and ATP gain

Microorganisms carry out catabolic oxidation–reduction reactions to obtain energy for growth and cell maintenance. These energy usages are captured in a genome-scale model in the form of two parameters, growth associated maintenance (GAM mmolATP/gDCW) and non-growth associated maintenance (NGAM mmolATP/gDCW/h). GAM represents the energy required for the polymerization of macromolecules, such as DNA, RNA, proteins, and glycogen, during growth. It appears as the stoichiometric coefficient of ATP in the reaction representing biomass formation (cell growth) in *i*MM518. In contrast, NGAM represents the energy (mmolATP/gDCW/h) required for cell repair, motility, maintenance of ion gradients etc., which the cell uses in addition to GAM [21, 22].

While it is possible to theoretically estimate GAM, it is not possible to estimate NGAM. Using a literature procedure [23], we estimated GAM as 30.0 mmolATP/gDCW for *M. maripaludis*. Although literature did not report any values for GAM and NGAM for *M. maripaludis*, we were able to estimate them using *i*MM518 and with our comprehensive experimental data in this study. Our earlier validation of *i*MM518 was based on limited biomass growth data and phenotypic observations on gene knock-outs due to the unavailability of experimentally measured fluxes [13]. In this study, we are presenting a novel procedure to estimate GAM and NGAM precisely for *M. maripaludis*, and validating our model predictions for extracellular fluxes.

**Fig. 4:**
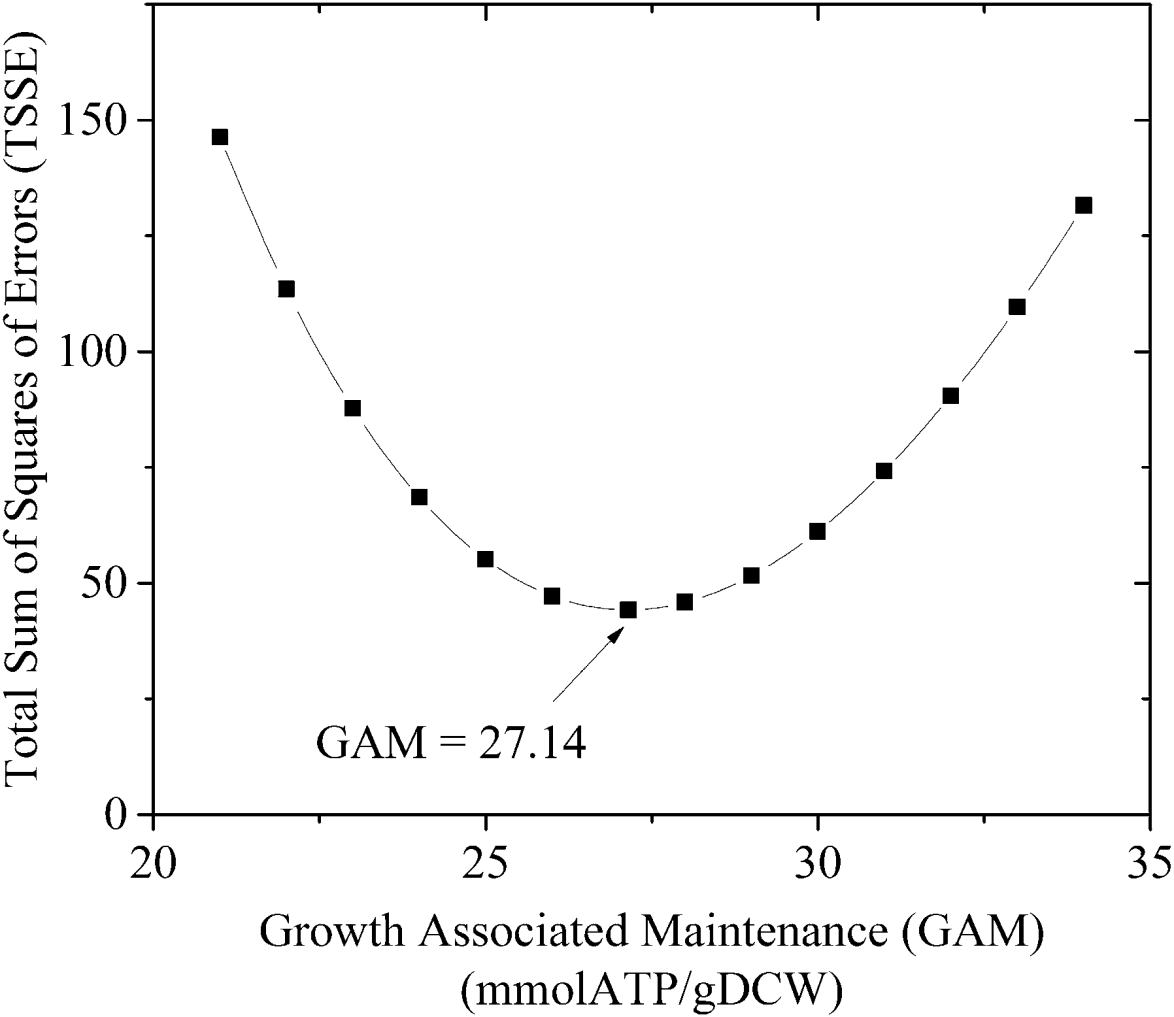
The variation of TSSE with GAM with the minimum at GAM = 27.14 mmol/gDCW for NGAM = 7.836 mmol/gDCW/h. *NGAM* is non-growth associated maintenance energy

Our experiments indicated that cell growth rate was zero at a CO_2_ uptake of 23.51 mmol/gDCW/h, and the non-growth maintenance energy that gave zero growth prediction from *i*MM518 was 7.836 mmolATP/gDCW/h. Thus, NGAM was calculated as 7.836 mmolATP/gDCW/h. NGAM was then fixed in the model and the total Sum of Squares of Errors (TSSE) was calculated for a range of GAM values. Figure 4 shows how TSSE varied with GAM. TSSE was minimum at GAM = 27.14 mmolATP/gDCW, which is the best estimate of GAM from our experiments. The deviations in H_2_ uptake predictions contributed the most (67.4 %) to the minimum TSSE = 0.044, followed by those in CH_4_ evolution rates (31.7 %). The prediction of biomass growth from the model matched very well with the experimental values. Model predictions of growth rates showed less than 1 % (0.76 %) deviations from measured values. Our estimated GAM of 27.14 mmolATP/gDCW/h agrees very well with the theoretical estimate of 30.0 mmolATP/gDCW/h) [13] for *M. maripaludis*. Table 2 lists GAM and NGAM reported for selected microorganisms in the literature.

**Table 2:** Comparison of our estimated GAM and NGAM for *M. maripaludis* with those reported for other model organisms

Organism	Substrate	GAM (mmol/gDCW)	NGAM (mmol/gDCW/h)	References
*Escherichia coli*	Glucose	59.81	8.39	[35]
*Lactococcus lactis*	Different sugar substrates e.g. mannose, galactose, sucrose, lactose, etc.	18.15	1	[36]
*Methanosarcina barkeri*	Methanol or acetate or H_2_/CO_2_ or pyruvate	70	1.75	[15]
*Methanosarcina acetivorans*	CO or methanol	65	2.5	[37]
*Methanococcus maripaludis*	H_2_/CO_2_	27.14	7.836	This study

*GAM* growth associated maintenance energy, *NGAM* non-growth associated maintenance energy

A common method for estimating GAM and NGAM is to measure substrate uptake fluxes (mmol substrate/gDCW/h) at different growth rates and use an estimated ATP gain (mol ATP/mol substrate). However, the difficulty with this approach is that precise values for ATP gains are unavailable for most microbes including *M. maripaludis*, as it is difficult to assess the amount of ATP generation per mole of substrate or product. In contrast, our approach combines experimentally measured values along with a genome-scale model to estimate NGAM and GAM without requiring an ATP gain. In fact, we estimated ATP gains from our NGAM as 0.33 mol ATP/mol CO_2_, 0.35 mol ATP/mol CH_4_, and 0.238 mol ATP/mol H_2_. While the ATP gains from CO_2_ and CH_4_ are similar, the ATP gain from H_2_ is much lower. This could be due to the deviations observed in our flux predictions for H_2_ in TSSE. The value of 0.35 molATP/molCH_4_ is in the acceptable range of 0.3–0.7 mol ATP/molCH_4_ reported for microbes with autotrophic growth on H_2_/CO_2_ [24]. Kaster et al. [25] suggested an ATP gain of less than 1 mol ATP/mol CH_4_ for methanogens without cytochromes (e.g. *M. maripaludis*) and more than 1 mol ATP/mol CH_4_ for methanogens with cytochromes (e.g. *M. barkeri*). Thus, our estimate of 0.35 mol ATP/mol CH_4_ is in agreement with the literature.

**Fig. 5:**
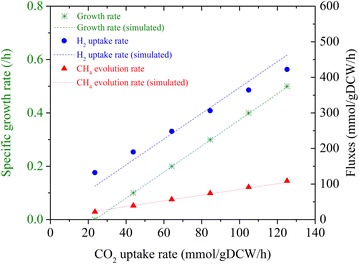
Comparison of model predicted growth rates, H_2_ uptake rates, and CH_4_ evolution rates with experimentally measured values for fixed CO_2_ uptake rates

With GAM = 27.14 mmol/gDCW and NGAM = 7.836 mmol/gDCW/h in *i*MM518, we fixed CO_2_ uptake rate at various values and predicted cell growth, MER, and H_2_ uptake rate for the maximum biomass growth. Figure 5 compares experimental results with our model predictions. As we can see, our model predictions and experimental results match very well.

### Intracellular fluxes

Genetic and/or environmental perturbations can change extracellular or intracellular fluxes in an organism. If a genome-scale model can be used to simulate these perturbations, then it can help us study phenotypes under various culture conditions, improve microbial strains in bioprocesses, analyze multispecies relationships, etc. Therefore, we further applied *i*MM518 to study the impacts of various experimentally studied or hypothetical scenarios on the distribution of intracellular fluxes in *M. maripaludis* S2.

### Effects of amino acids and vitamins from *i*MM518

The amino acids are known to stimulate the growth of autotrophic methanococci [26, 27], but the precise mechanisms behind these stimulations and the effects of amino acids on methanogenesis are unknown. To study these, we first modeled a culture with all amino acids. We fixed CO_2_ uptake at 60 mmol/gDCW/h in *i*MM518, and allowed unlimited uptakes for all amino acids. As compared to the scenario with no amino acids in the culture, the cell growth increased by 44.4 %, and MER increased by 11.2 %. The former is consistent with previous experimental observations at least qualitatively [26, 27], while the latter has not been measured in the literature. *M. maripaludis* prefers alanine overwhelmingly over all other amino acids, as the uptake distribution was alanine 34.6 mol%, aspartate 14.4 mol%, serine 13.7 mol%, leucine 5.8 mol%, valine 4.6 mol%, and the rest <5.0 mol%. *M. maripaludis* avoids the uptake of glycine, tyrosine, glutamate and glutamine.

**Fig. 6:**
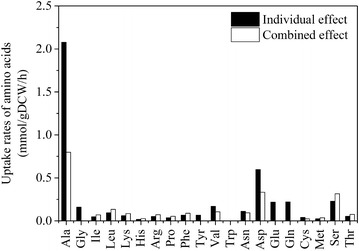
In silico uptake rates of amino acids when supplied individually versus all-together in the culture medium

In order to study the effects of amino acids individually, we performed 20 culture simulations with single amino acid each in *i*MM518. Interestingly, the uptakes and effects were quite different from what we observed with all amino acids in one culture simulation (Fig. 6). This could be primarily because the microbe prefers some amino acids over others for energetic reasons. Table 3 lists the changes in cell growth and MER due to each amino acid. Alanine proved the most effective for growth and MER, as it increased growth by 11.4 % and MER by 10.3 %. In contrast to the earlier scenario with all amino acids, the microbe consumed aspartate, and growth increased by 11.5 % and MER by 3.0 %. All other amino acids individually increased growth rate by less than 7 %. To evaluate the differences in the increments of growth and MER between alanine and aspartate, we examined the distribution of intracellular fluxes in both cases. Alanine served as the sole nitrogen source which has been confirmed by previous experiments [26]. On the other hand, aspartate could not supply the entire nitrogen demands of the cell, but acted as a supplement to reduce ammonium uptake by 25.9 %. Alanine increased growth primarily by supplying additional pyruvate via the reaction alanine + NAD + H_2_O ↔ pyruvate + NH_3_ + NADH + H, which in turn increased the biosynthesis of cell growth precursors such as amino acids. Most of the CO_2_ was diverted to methane production to provide the energy for the additional growth, and hence MER also increased. The model also predicted that the autotrophic formation of acetyl-CoA drastically reduced (approx. 65.4 %) with alanine in medium. This is consistent with previous experimental results that alanine is an efficient means of labeling pyruvate, as only 3–5 % of the carbon in acetyl-CoA was from CO_2_ [28]. On the other hand, aspartate did not contribute significantly towards the formation of pyruvate and only 10 % drop in the flux was observed for the formation of acetyl-CoA from CO_2_.

**Table 3:** Individual and combined effects of amino acids on growth rates, H_2_ uptake rates, and CH_4_ evolution rates as predicted by *i*MM518

Amino acids	% increment in specific growth rates	% increment in H_2_ uptake rates	% increment in CH_4_ evolution rates
Alanine	11.38	4.82	10.25
Glycine	2.19	0.22	0.31
Isoleucine	0.79	0.14	0.44
Leucine	1.54	0.28	0.86
Lysine	1.97	0.15	0.41
Histidine	1.50	0.02	−0.01
Arginine	2.25	−0.09	−0.21
Proline	0.30	−0.05	−0.04
Phenylalanine	6.52	0.28	0.61
Tyrosine	6.52	0.31	0.61
Valine	2.32	0.46	1.29
Tryptophan	0.14	0.00	0.01
Asparagine	3.06	0.31	0.43
Aspartate	11.46	1.95	2.95
Glutamate	0.00	0.00	0.00
Glutamine	1.82	−0.11	−0.25
Cysteine	0.55	0.09	0.15
Methionine	0.46	0.06	0.12
Serine	3.74	0.48	0.78
Threonine	1.89	0.07	0.14
Combined	44.39	11.17	5.4

Interestingly, several amino acids such as arginine, histidine, proline and glutamine showed marginally reduced (<0.1 %) MERs. This is because the cell saved the energy for making these amino acids. Methanogenesis, being the only energy producing pathway in *M. maripaludis*, reduced MER accordingly. Our in silico study showed that none of the vitamins including water-soluble riboflavin, biotin, and vitamin B12 affected growth at all. This is again validated by a previous experimental study [26], and needs no repeat experimental validation.

## Conclusions

Our experimental measurements of extracellular fluxes were in excellent agreement with in silico predictions of *i*MM518 at GAM = 27.14 mmol/gDCW and NGAM = 7.836 mmol/gDCW/h, thus allowed us to elucidate the physiological and metabolic states of the cells during batch culture. With *M. maripaludis*, an instantaneous conversion of 85–95 % from CO_2_ to CH_4_ was observed at 37 °C, while the conversion on a metal catalyst did not go beyond 70 % even at 800 °C [1]. Understanding biofuel production from methanogens will help scientists develop a bioreactor employing immobilized enzymes instead of a whole cell bioreactor. It is also possible to perform methanogenesis from CO_2_ at a very fast rate and avoid wasteful microbial biomass without the need for biofuel extraction.

Biochemical conversion of CO_2_ to biofuels using such strategies provides a promising route for more efficient renewable energy production. It should be noted that the cellular objective could be different depending on environmental or physiological conditions, with considerable implication on the final cellular phenotype [29]. For example, maximizing the growth rate during stationary phase may not be cellular objective. In that case it is important to identify the most plausible cellular objectives, such as minimization of ATP production, maximization of metabolite production, and minimization of nutrient uptake, and the predictive power a genomic-scale model can be greatly improved.

## Methods

### Chemicals and gases

All chemicals used in this study were American Chemical Society (ACS) analytical reagents purchased from Sigma-Aldrich. Pure gases (N_2_, Ar) and 80/20 v/v H_2_/CO_2_ mixture were purchased from AIR Liquide, Singapore.

### Strain and medium

*Methanococcus maripaludis* S2 (DSM 14266) was purchased from DSMZ-German Collection of Microorganisms and Cell Cultures. Methanococcus culture medium 141 was used to cultivate the culture at 37 °C with a headspace pressure of 200 kPa under 80/20 H_2_/CO_2_ and constantly stirred at 180 rpm [30]. The minimal medium for the growth experiments comprised 0.34 g of KCl, 4 g of MgCl_2_·6H_2_O, 3.45 g of MgSO_4_·7H_2_O, 0.25 g of NH_4_Cl, 0.14 g of CaCl_2_·2H_2_O, 0.14 g of K_2_HPO_4_, 18 g of NaCl, 10 mL of trace elements, and 2 mg of Fe(NH_4_)_2_(SO_4_)_2_·7H_2_O per liter. The trace element solution comprised 3 g MgSO_4_·7H_2_O, 0.5 g MnSO_4_·H_2_O, 1 g NaCl, 0.10 g FeSO_4_·7H_2_O, 0.18 g CoSO_4_·7H_2_O, 0.10 g CaCl_2_·2H_2_O., 0.18 g ZnSO_4_·7H_2_O, 0.01 g CuSO_4_·5H_2_O, 0.02 g KAl(SO_4_)_2_·12H_2_O, 0.01 g H_3_BO_3_, 0.01 g Na_2_MoO_4_·2H_2_O, 0.03 g NiCl_2_·6H_2_O, 0.30 mg Na_2_SeO_3_·5H_2_O, and 990 mL DI H_2_O. Soluble carbon source and cysteine were removed and CO_2_ was the only carbon source. Vitamins were also omitted [26].

### Batch cultivation

230 mL of medium was dispensed into 600 mL serum bottles, and sparged with 80/20 v/v H_2_/CO_2_ to remove dissolved oxygen and create an anaerobic atmosphere. After autoclaving at 121 °C for 20 min, the bottles were cooled to room temperature and 0.5 mg/mL of Na_2_S·7H_2_O was injected. To initiate the growth in minimal medium, 20 mL of inoculum (pre-cultured cells in late exponential phase) was injected into each bottle. The bottles were then pressurized with 250 kPa 80/20 v/v H_2_/CO_2_ and incubated at 37 °C under constant stirring at 180 rpm. Cell density and concentrations of CO_2_, H_2_, and CH_4_ in the headspace were measured. The growth experiments were discontinued when the headspace pressure fell below 100 kPa to avoid the inflow of air into the reactor. All growth experiments were performed in duplicates accompanied by a control experiment with no inoculum.

### Analytical procedures

Cellular growth was monitored by measuring optical density (OD) of 1 mL culture samples during the experiments. OD was recorded at 600 nm using a double-beam UV/Vis Spectrophotometer (Hitachi Model U-2800, High Technologies America, Inc.). Our OD measurements had a standard deviation of 3.35 × 10^−3^. Bottle pressure was measured using a M1 digital pressure gauge (Cole Parmer, USA) with sensitivity of 10^−4^ bar. Headspace gases were analyzed with an Agilent 7890A series SRI Instrument GC equipped with three columns (a Porapak Q 80/100 SS packed column of size 6 ft L × 1/8″ OD × 2 mm ID, a Molecular Sieve 5A 80/100 SS packed column of size 3 ft L 1/8″ OD 2 mm ID, and a Hayesep T 80/100 UM column of size 0.5 m L 1/8″ OD 2 mm ID) and a thermal conductivity detector with electronic pneumatic control (EPC). The carrier gas (Ar) was continuously supplied at 100 psig. N_2_ was supplied at 30 psig to act as the actuation gas to compensate for the pressure and volume differences between the injected sample and required standard. The GC oven was maintained at 60 °C and front detector at 150 °C. 1 mL of gas samples were drawn from the bottles using gas airtight microsyringes (Hamilton Samplelock syringe), and analyzed immediately in GC. The GC was calibrated for dry gas compositions (% v/v) using the series of standards.

### Cell growth measurements

To estimate specific growth rate (µ), OD was measured at various time points. Lupa et al. [6] have reported an experimentally measured value (1 *OD*_600 nm_ = 0.34 g DCW/*L*) for converting OD to dry cell biomass specifically for *M. maripaludis S2*. Using this, the measured OD values were converted to biomass given by X gDCW = OD × 0.34 g/L × culture volume and specific growth rate (dX/dt)/X was computed by curve-fitting and differentiating the time profile.

### Calculation of extracellular fluxes

**Fig. 7:**
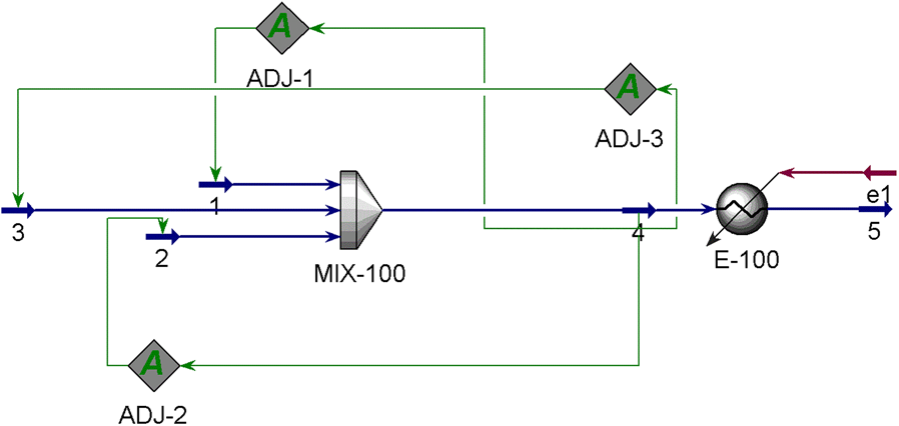
Block flow diagram in Aspen Hysys for simulating the dynamics of a batch reactor

Estimating extracellular fluxes from a cell culture study is not straightforward as gases are distributed into both aqueous media and headspace. The fraction of gases in the aqueous medium depends on bottle temperature, pressure, mixing speed, and the solubility and dissociation properties of the gases in water. In order to estimate fluxes precisely, we simulated the dynamics of a 600 mL reactor using Aspen HYSYS V8.2 [31] for the entire experiment. The block flow diagram for this simulation is shown in Fig. 7. This method can also be used for all hydrogenotrophic methanogens that can grow on CO_2_ as the sole carbon substrate.

We simulated this initial state of the bottle by mixing sufficient amounts of pure water (stream 2), 80/20 H_2_/CO_2_ (stream 1), and pure CO_2_ (stream 3). The flows of streams 1 and 2 were adjusted to achieve 350 mL headspace and 250 mL liquid medium, while the flow of stream 3 was adjusted to make the amount of CO_2_ in the headspace exactly equal to that supplied in stream 1. We measured culture OD, bottle pressure, bottle temperature, and headspace gas compositions. At each time point, we set stream 1 composition to be the same as the headspace composition (dry basis), the temperatures of streams 1, 2, and 3 as 37 °C, and the pressures of streams 1, 2, and 3 using the measured pressure. Then, we computed the total moles of H_2_ and CH_4_ in the bottle at each time point as the sum of moles of H_2_ (CH_4_) in the headspace from Hysys and moles of H_2_ (CH_4_) in the aqueous medium from Hysys. For computing the total moles of CO_2_ in the bottle, we also accounted for the high solubility (1.05 g/L) and dissociation of CO_2_ into bicarbonates (HCO_3_^−^) and carbonates (CO_3_^2−^). The initial pH of the growth culture was measured as 5.6, and it remained constant throughout the experiment (data not shown). Therefore, [H^+^] was fixed at 10^−5.6^ for the above calculations. Then, [H_2_CO_3_] obtained from Aspen Hysys was used to compute other ionic concentrations. The total amount of CO_2_ at time *t* was computed as the sum of *n*(CO_2_) from Aspen Hysys, HCO_3_^−^, and CO_3_^2−^. The fluxes $v_{i} = {1 \mathord{\left/ {\vphantom {1 {X(t)}}} \right. \kern-0pt} {X(t)}} \cdot {{dn(i)} \mathord{\left/ {\vphantom {{dn(i)} {dt}}} \right. \kern-0pt} {dt}}$ for CO_2_ and H_2_ consumption and CH_4_ production were computed by plotting the time profiles of total moles of CO_2_, H_2_, and CH_4_, where *n(i)* is the moles of species *i*(CO_2_, H_2_, or CH_4_) in the bottle and *X(t)* is the dry cell mass (g DCW) at time *t*.

### Parameter estimation for iMM518

*i*MM518 is a genome-scale in silico metabolic model developed for *M. maripaludis* and is available in BioModel database as MODEL1304120000 [13]. It comprises 570 reactions and 556 distinct metabolites, and covers 518 (~30 %) of the known 1722 open reading frames (ORFs). We implemented *i*MM518 in GAMS (build 38380/38394) and used CPLEX and BARON, respectively as the solvers for various linear and nonlinear optimization problems. *i*MM518 uses two energy parameters: GAM and NGAM. We showed how the experimental data on fluxes and growth can be integrated with an in silico model (*i*MM518 in this case) to estimate GAM and NGAM. First, we used the time profiles of extracellular fluxes and specific growth rates to obtain a regression for each flux with growth rate, and estimated experimental growth rates for various CO_2_ uptake rates.

For flux balance analysis, we assumed the cellular objective to be maximum biomass. Then, to predict cell growth rate for a given CO_2_ uptake rate, we solved the following linear programming (LP) using *i*MM518.

3 $${\text{Maximize}}\;Z = \sum\nolimits_{j = 1}^{J} {c_{j} v_{j} } \;{\text{subject to}}\;S \cdot v = b$$

where, Z is the cellular objective that is represented as a weighted sum of metabolite fluxes $v_{j} (j = 1,2, \ldots ,J)$ with weights $c_{j}$, $S$ is an $I \times J$ matrix of stoichiometric coefficients of the metabolic reactions, $I$ is the number of metabolites, $J$ is the number of metabolic reactions, $v$ is a $J \times 1$ vector of reaction fluxes, and *b* is a $I \times 1$ vector of net metabolite fluxes.

The linear CO_2_ flux vs growth rate relationship reported above showed that cell growth was zero for a CO_2_ uptake rate below 23.51 mmol/gDCW/h, and we computed NGAM as the amount of energy spent for maintenance without growth. For estimating GAM, we selected twelve CO_2_ uptake rates ($v_{{{\text{CO}}_{ 2} }}^{k} = b_{{{\text{CO}}_{ 2} }}^{k} ,\quad k = 1,2, \ldots ,12$). Using the CO_2_ flux vs growth rate expression, we computed respective experimental growth rates ($b_{G}^{k} ,\quad k = 1,2, \ldots ,12$). We estimated the experimental fluxes ($b_{{{\text{CH}}_{ 4} }}^{k}$ and $b_{{{\text{H}}_{ 2} }}^{k} ,\quad k = 1,2, \ldots ,12$) for CH_4_ and H_2_ at these $b_{G}^{k}$ using the linear expressions for CH_4_ and H_2_ fluxes vs growth rates. Further, we fixed the CO_2_ uptake rates inside *i*MM518 to predict cell growth rates ($v_{G}^{k} ,\quad k = 1,2, \ldots ,12$) and fluxes for CH_4_ ($v_{{{\text{CH}}_{ 4} }}^{k}$) and H_2_ ($v_{{{\text{H}}_{ 2} }}^{k}$) for varying values of GAM. For each GAM value, we used these model predictions to compute a weighted sum of squares of errors (SSE) as follows:

4 $$SSE(GAM) = \sum\nolimits_{k = 1}^{12} {[\gamma_{G}^{2} } (v_{G}^{k} - b_{G}^{k} )^{2} + \gamma_{{{\text{CH}}_{ 4} }}^{2} (v_{{{\text{CH}}_{ 4} }}^{k} - b_{{{\text{CH}}_{ 4} }}^{k} )^{2} + \gamma_{{{\text{H}}_{ 2} }}^{2} (v_{{{\text{H}}_{ 2} }}^{k} - b_{{{\text{H}}_{ 2} }}^{k} )^{2} ]$$

where, γ_G_ = 3 gDCW/g DCW, γ_CH4_ = 0.016 g/mmol, and γ_H2_ = 0.002 g/mmol. The GAM value with the minimum SSE was our best estimation for the growth energy required by *M. maripaludis*.
